# The complete chloroplast genome sequence of *Chrysojasminum subhumile* and its phylogenetic position within Oleaceae

**DOI:** 10.1080/23802359.2023.2224460

**Published:** 2023-06-18

**Authors:** Xinyu Ling, Rui Liao, Xingfu Zhu

**Affiliations:** School of Life Sciences, Guizhou Normal University, Guiyang, China

**Keywords:** *Chrysojasminum subhumile*, chloroplast genome, phylogenetic analysis

## Abstract

We assembled and characterized the complete chloroplast genome sequence of *Chrysojasminum subhumile* (W.W.Sm.) Banfi & Galasso 2014, a valuable horticultural and medicinal plant species. The total genome size was 159,918 bp in length and the GC content was 37.4%. It displayed a circular structure and could be divided into a large single-copy region, a small single-copy region, and a pair of inverted repeat regions. The genome encoded a total of 131 unique genes, including 82 protein-coding genes, 41 tRNA genes, and eight rRNA genes. Among these genes, 17 contained a single intron, and two genes had two introns. Phylogenetic analysis results showed that *C. subhumile* was closely related to *Jasminum*.

## 1. Introduction

*Chrysojasminum* is a genus of flowering plants in the family Oleaceae and order Lamiales. Oleaceae plants are widely distributed in tropical and temperate regions of both hemispheres, with a particularly rich variety in Asia. The Oleaceae family has many important medicinal plants, spice plants, oil plants, and economic tree species. *Chrysojasminum* was established to revise the generic delimitation of jasmine (Banfi 2014), since those species possessing both alternate leaves and yellow flowers constitute a monophyletic group that is sister to all other jasmine plus the American/South African genus *Menodora* (Lee et al. 2007; Kim and Kim 2011).

**Figure 1.**
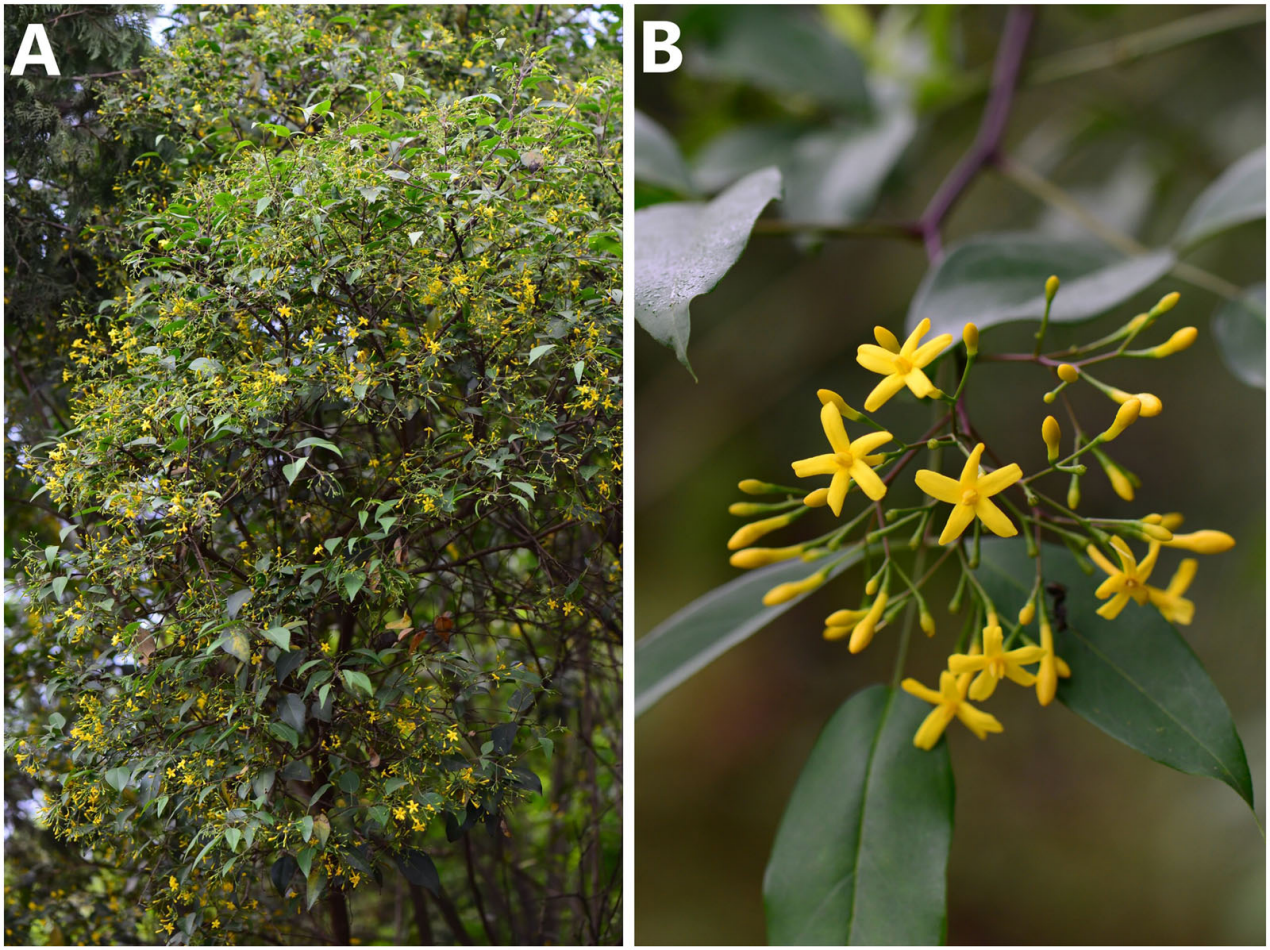
The species reference image for *Chrysojasminum subhumile*. (A) A crow of *C. subhumile*; (B) a young branch of *C. subhumile* with yellow flowers and alternate leaves; the photos were taken by RenBin Zhu of Xishuangbanna Tropical Botanical Garden.

The chloroplast is a crucial organelle for photosynthesis, as well as vitamin, starch, protein, and pigment synthesis. Chloroplast genes are generally matrilineal in angiosperms, with only a few species exhibiting biparental or paternal inheritance (Hu 2008). As the chloroplast genome is the smallest genome in plant cells, with easily accessible full sequences, modest evolution rate, and conserved gene composition, it has become an ideal model for evolutionary and phylogenetical studies (Shaw et al. 2007; Mehmood et al. 2020a, 2020b, 2020c). As a consequence, the *Chrysojasminum* genomic data are required to examine its phylogenetic position within jasmine. In this experiment, we published the first comprehensive plastome report for *Chrysojasminum subhumile* (W.W.Sm.) Banfi & Galasso, a common ornamental and medicinal plant (Figure 1) (Chang et al. 1996). The annotated genomic sequence has been deposited in GenBank as registration number OK236384.

## 2. Materials and methods

Plant samples were collected from Kunming Institute of Botany, Kunming, China (E102.7515°, N25.1411°). Voucher herbarium specimens (zhu20200702) were stored in Guizhou Normal University’s Department of Botany (Xingfu Zhu, email: zhuxingfu@outlook.com). Using the Qiagen DNeasy Plant Mini Kit (Qiagen, Carlsbad, CA), we extracted the whole DNA from one *C. subhumile* sample, and then we carried out the subsequent high-throughput sequencing on an Illumina Hiseq 2500 System. The raw data after filtered with Trimmomatic 0.38 (Bolger et al. 2014) were delivered to the GetOrganelle program (Jin et al. 2020) to build the whole chloroplast genome, the usage parameters are: -R 15 -k 21,45,65,85,105 -F embplant_pt. The CPGview (Liu et al. 2023) and PGA software (Qu et al. 2019) were used to perform the genome annotation with default parameter.

## 3. Results and discussion

### 3.1. Genome compositions

**Figure 2.**
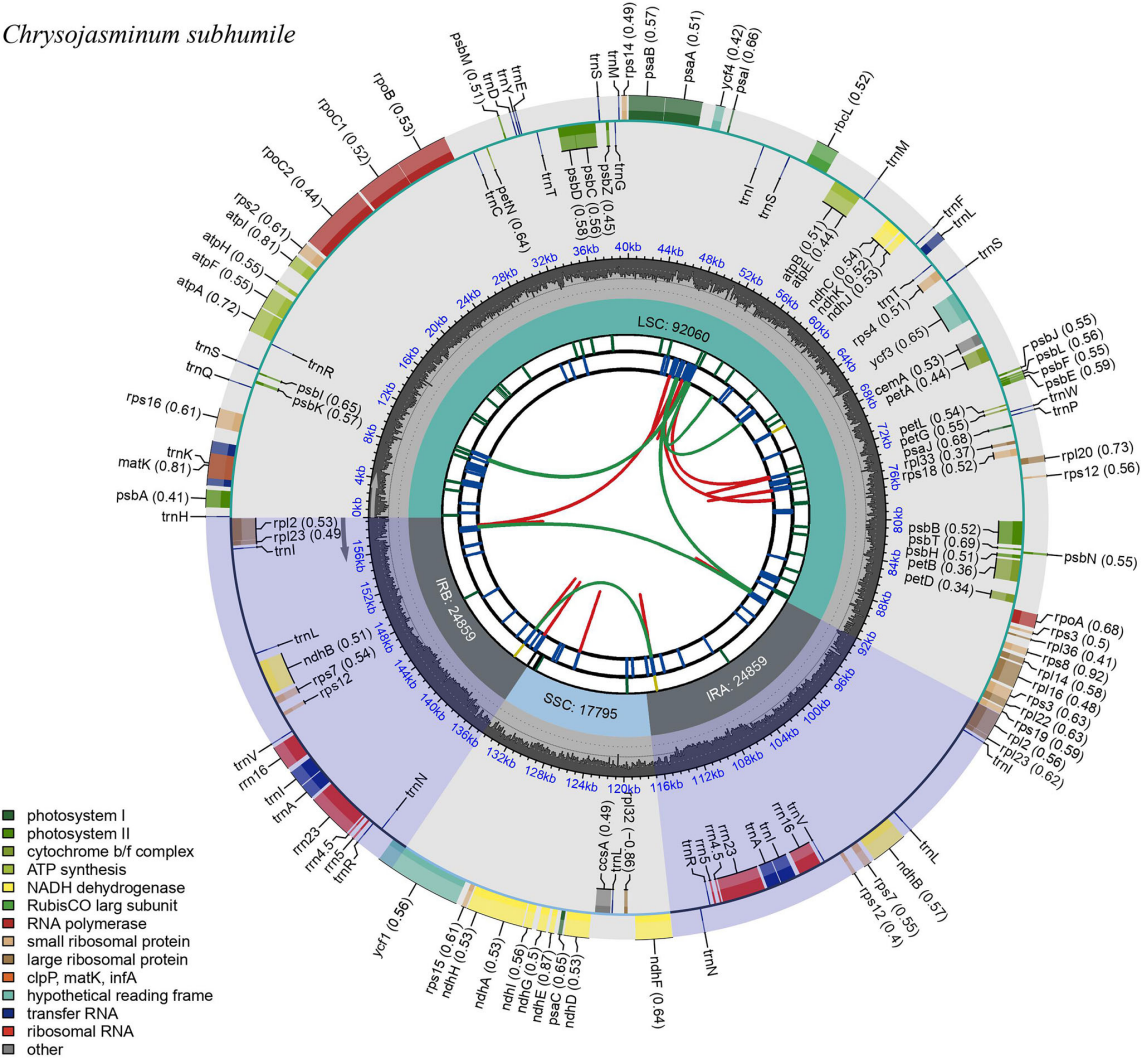
Graphic representation of features identified in *Chrysojasminum subhumile* chloroplast genome using cpgview (http://www.1kmpg.cn/cpgview). The map contains six rings. From the center outward, the first track shows the dispersed repeats. The dispersed repeats consist of direct (D) and Palindromic (P) repeats, connected with red and green arcs. The second track shows the long tandem repeats as short blue bars. The third track shows the short tandem repeats or microsatellite sequences as short bars with different colors. The colors, the type of repeat they represent, and the description of the repeat types are as follows. Black: c (complex repeat); green: p1 (repeat unit size = 1); yellow: p2 (repeat unit size = 2); purple: p3 (repeat unit size = 3); blue: p4 (repeat unit size = 4); orange: p5 (repeat unit size = 5); red: p6 (repeat unit size = 6). The small single-copy (SSC), inverted repeat (IRa and IRb), and large single-copy (LSC) regions are shown on the fourth track. The GC content along the genome is plotted on the fifth track. The base frequency at each site along the genome will be shown between the fourth and fifth tracks. The genes are shown on the sixth track. The optional codon usage bias is displayed in the parenthesis after the gene name. Genes are color-coded by their functional classification. The transcription directions for the inner and outer genes are clockwise and anticlockwise, respectively. The functional classification of the genes is shown in the bottom left corner.

The chloroplast genome of *C. subhumile* has a typical cyclic tetrad structure, which consists of a pair of 23,022 bp repetitive sequences and two single copy regions with 95,780 bp and 17,794 bp, respectively (Figure 2). It encodes 131 distinct genes in total, 13 of which are duplicated in the IR areas. It has eight rRNA genes, 41 tRNA genes, and 82 protein-coding genes. In addition, 11 genes only contain one intron, while one gene (ycf1) has five intros and one gene (ycf3) has two introns.

### 3.2. Comparative analysis of chloroplast genomes

**Figure 3.**
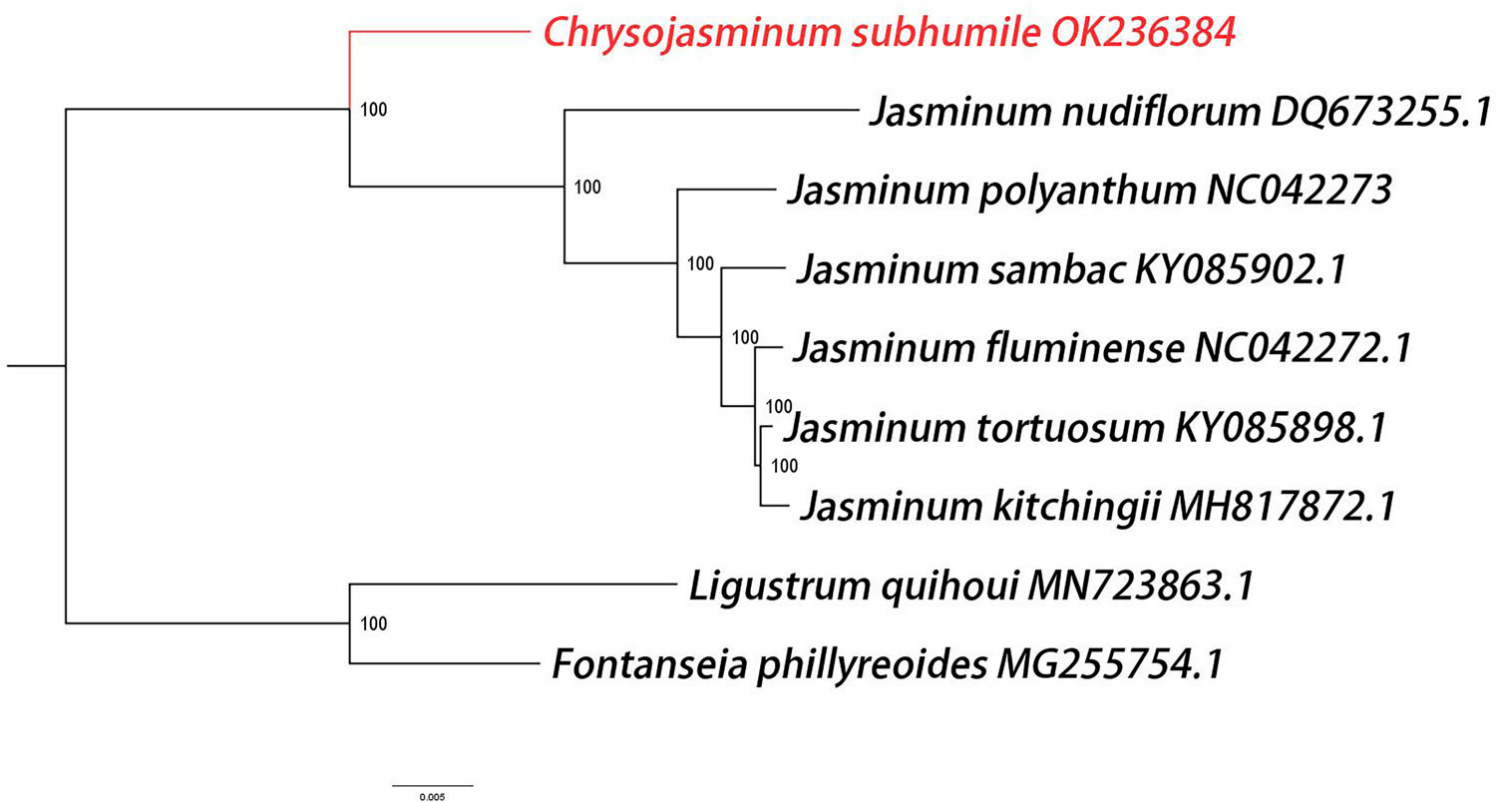
Phylogenetic relationships among nine complete chloroplast genomes of Oleaceae. The newly reported genome of *Chrysojasminum subhumile* was represented in red. *Fontanesia phillyreoides* and *Ligustrum quihoui* of Oleaceae family were taken as outgroups. Bootstrap support values are given at the nodes and the GenBank accession number was listed after the species name.

In order to determine the phylogenetic position of *C. subhumile*, six complete chloroplast genomes of *Jasminum* were obtained from the GenBank. In addition, *Fontanesia phillyreoides* and *Ligustrum quihoui* of Oleaceae family were taken as outgroups. Seventy chloroplast protein-coding genes were extracted from the nine complete chloroplast sequences, and then they were compared by MAFFT 7th edition software (Katoh and Standley 2013). The phylogenetic analysis was performed on the maximum-likelihood analysis of RAxML under the general time reversible nucleotide substitution model with the gamma model of rate heterogeneity (Alexandros 2014). The findings are consistent with previous researches that the phylogenetic analysis strongly supported *C. subhumile* as a sister species of *Jasminum* (Lee et al. 2007; Kim and Kim 2011) (Figure 3). The sequenced chloroplast genome of C. *subhumile* will be an important genetic resource for further research on the genetic diversity and conservation of jasmine plants.

## Supplementary Material

Supplemental Material

Supplemental Material

Supplemental Material

Supplemental Material

## Data Availability

The genome sequence data that support the findings of this study are openly available in GenBank of NCBI at https://www.ncbi.nlm.nih.gov/ under the number OK236384. The associated BioProject, SRA, and Bio-Sample numbers are PRJNA842199, SRR19448979, and SAMN28649230, respectively.
