# Supplementary figures and images for: The complete chloroplast genome sequence of Chrysojasminum subhumile and its phylogenetic position within Oleaceae
Source: Mitochondrial DNA B Resour. 2023 Jun 18;8(6):678–81. doi: 10.1080/23802359.2023.2224460 (PMC10281350; doi:10.1080/23802359.2023.2224460)

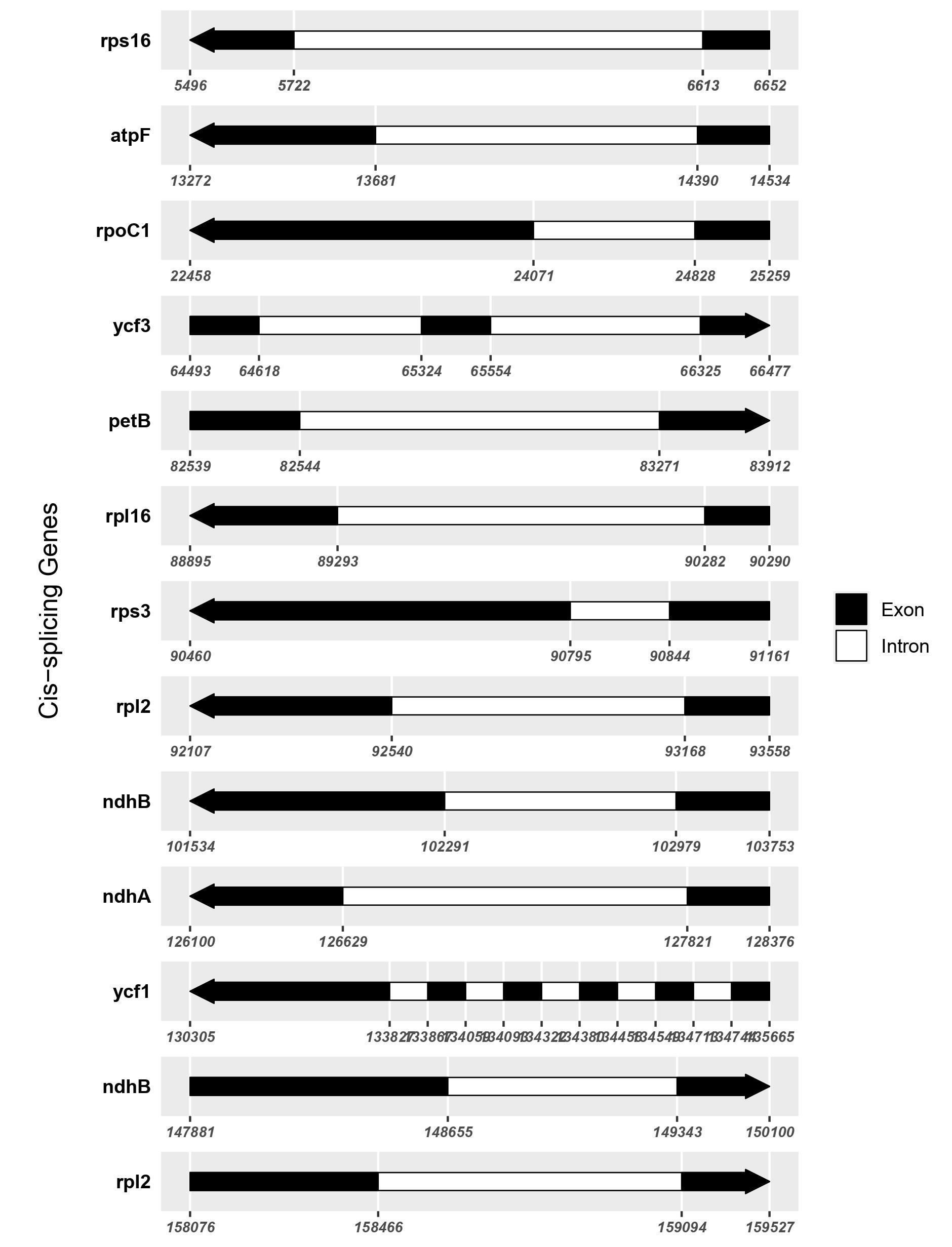

Supplement: Supplemental Material [file TMDN_A_2224460_SM0481.jpg]

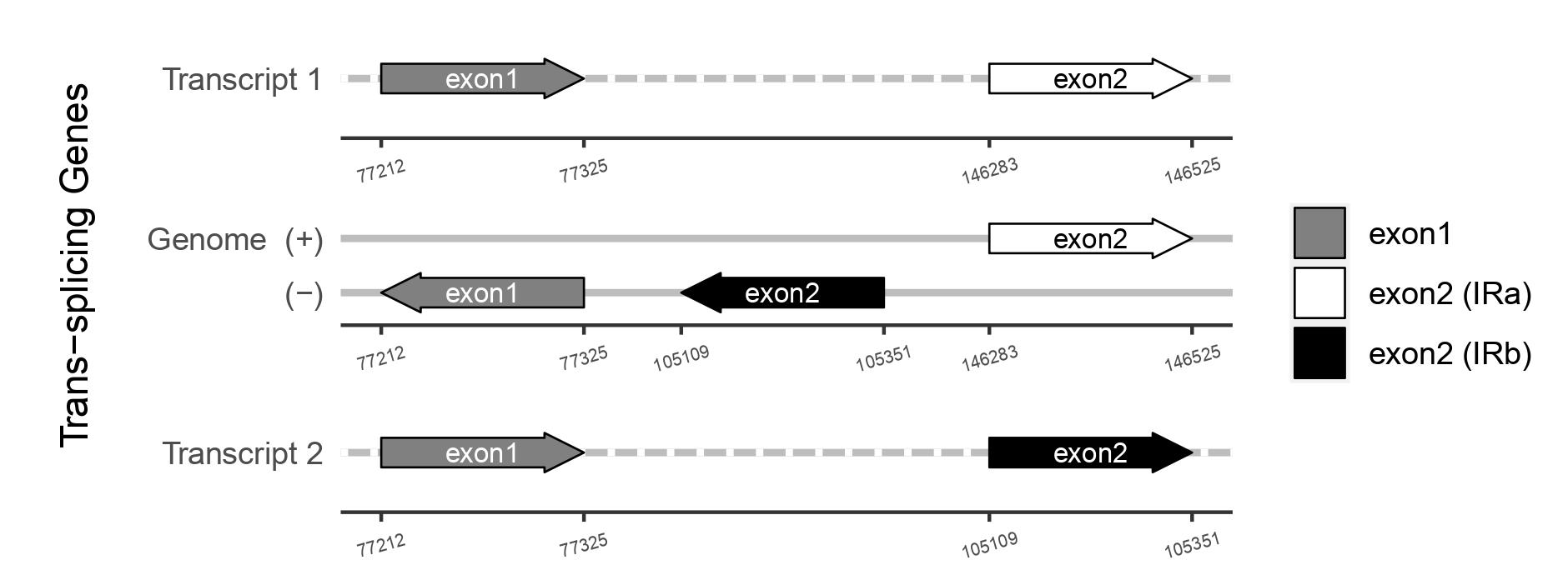

Supplement: Supplemental Material [file TMDN_A_2224460_SM0467.jpg]

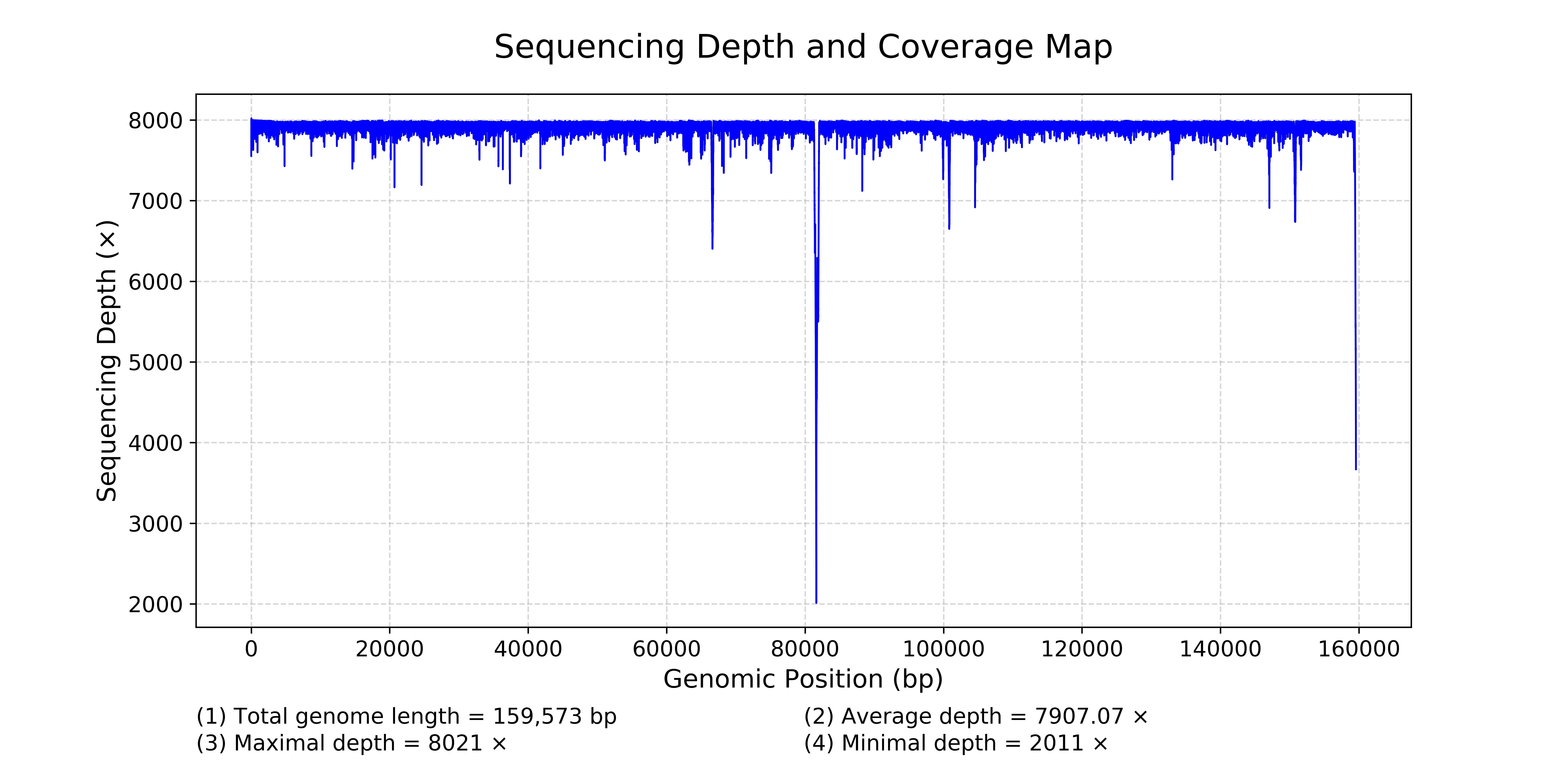

Supplement: Supplemental Material [file TMDN_A_2224460_SM0450.jpg]
